# Designing an Ultrathin Film Spectrometer Based on III-Nitride Light-Absorbing Nanostructures

**DOI:** 10.3390/mi12070760

**Published:** 2021-06-28

**Authors:** Juhyeon Kim, Srinivasa Cheekati, Tuba Sarwar, Pei-Cheng Ku

**Affiliations:** Department of Electrical Engineering and Computer Science, University of Michigan, 1301 Beal Ave, Ann Arbor, MI 48109, USA; juhyeon@umich.edu (J.K.); scheekat@umich.edu (S.C.); tsarwar@umich.edu (T.S.)

**Keywords:** gallium nitride, quantum confined Stark effect, strain control, compressive sensing

## Abstract

In this paper, a spectrometer design enabling an ultrathin form factor is proposed. Local strain engineering in group III-nitride semiconductor nanostructured light-absorbing elements enables the integration of a large number of photodetectors on the chip exhibiting different absorption cut-off wavelengths. The introduction of a simple cone-shaped back-reflector at the bottom side of the substrate enables a high light-harvesting efficiency design, which also improves the accuracy of spectral reconstruction. The cone-shaped back-reflector can be readily fabricated using mature patterned sapphire substrate processes. Our design was validated via numerical simulations with experimentally measured photodetector responsivities as the input. A light-harvesting efficiency as high as 60% was achieved with five InGaN/GaN multiple quantum wells for the visible wavelengths.

## 1. Introduction

Optical spectroscopy is a versatile technique in many branches of science and engineering. Miniaturized spectrometers and their array can enable a wide range of applications, including, but not limited to, health diagnostics, biochemical sensing, security, environmental science, planetary exploration, and lab-on-a-chip systems [1,2,3,4,5,6,7,8,9,10,11,12]. A standard spectrometer design utilizes diffractive optical elements (DOE) such as a diffraction grating to separate different spectral components into different optical paths for analysis. Most DOEs have a very narrow acceptance angle for the incident light, requiring additional optical elements to collimate or filter the input signal. Collimation optics typically consist of a narrow aperture, either a slit or pinhole, and a lens, adding to the system bulkiness and weight. A spectrometer design based on absorptive spectral parsing elements was recently proposed and demonstrated using both colloidal quantum dots and epitaxial nanostructures [4,13,14,15,16,17]. Because light absorption in nanostructures only weakly depends on the incident angle [18,19], an absorption-based spectrometer can enable a much more flexible and compact optical design, which benefits the realization of a spectrometer array for hyperspectral imaging or sensors with multiple modalities. Moreover, an optics-free spectrometer design, which opens up new opportunities such as wearable spectrometers for sweat sensor patches, also becomes possible.

An absorption-based spectrometer utilizes a series of optical filters with different absorption responses. The absorption responses from any two optical filters need to have minimal spectral correlations. Once this condition is satisfied, the photocurrent $I_{i}$ generated from the *i*-th photodetector/optical filter combination can be determined by

(1) $$I_{i}=R_{ij}\times P_{j}$$

where $P_{j}$ is the optical power at a wavelength $\lambda_{j}$ absorbed by the photodetector, and $R_{ij}$ is the responsivity, with a unit of A/W, of the *i*-th photodetector/optical filter combination at a wavelength $\lambda_{j}$. Once the responsivities of individual detection elements are measured, one can invert the above equation to obtain the optical spectrum *P*. In practice, the measured photocurrents include noises, and, thus, the above equation may not have a solution. However, one can seek a solution that minimizes the difference between the two sides of (1). This is a topic that has been the subject of immense interests in recent years [13,20,21,22,23,24]. As the spectra of interests are often not arbitrary, the accuracy of spectral reconstruction can be improved with a set of constraints, e.g., by assuming the optical spectrum consists of a series of Gaussian spectra with a finite linewidth. The estimation approach also allows one to recover important spectral information even when the number of photodetectors is less than the number of wavelengths [25].

The photodetector/optical filter combination can be further simplified by a photodetector design with an intrinsic tunable absorption response. For example, indium gallium nitride (InGaN) dot-in-the-wire (DIW) light-absorbing nanostructures have been shown to exhibit a tunable absorption response by changing their geometric parameters, such as diameter and shape [26]. The intrinsic strain stored in the InGaN DIW region grown on GaN due to lattice mismatch is relaxed near the surface of the nanostructure, modifying the electronic band structure and thus the absorption response. Lithographically defined InGaN DIW nanostructures can tune their absorption cut-off wavelengths from ultraviolet through near-infrared spectra [26,27,28,29]. Previously, an absorption-based spectrometer using an InGaN DIW nanostructure directly integrated with a GaN pn junction was demonstrated [17]. Such a design enables an extremely compact construction, eliminating the need for a separate silicon photodiode array. However, InGaN DIW nanostructures have very limited light absorption in the visible wavelength range due to a small absorption length. Significantly increasing the InGaN thickness will not be feasible due to a large lattice mismatch with GaN. This work proposes and demonstrates a simple strategy, utilizing the well-established sapphire substrate patterning process to greatly enhance the absorption efficiency while maintaining a large acceptance angle for the incident light. We also demonstrate that spectroscopic performance can be significantly enhanced.

## 2. Device Design

Although silicon is a ubiquitous and often preferred material for a wide range of electronic and optoelectronic applications, silicon’s light absorption properties cannot be easily tuned, which is necessary for an absorption-based spectrometer. To address this limitation, the proposed spectrometer design consists of an array of GaN-based photodiodes. Individual photodiodes’ absorption cut-off wavelengths are tuned by changing the nanopillar structures’ diameters. The underlying principle for the absorption tuning is local strain engineering [26]. When the compressive strain in the InGaN multiple quantum well (MQW) region is relaxed at the nanopillars’ sidewalls, the absorption bandedge is blue shifted due to the reduction of the quantum-confined Stark effect (QCSE). Local strain engineering has been previously applied to monolithically integrate red, green, and blue-light-emitting diodes (LEDs) on the same substrate using a single epitaxial stack [27,28,29,30]. The same principle has also enabled the realization of an absorption-based spectrometer [17]. InGaN absorbs light efficiently, exhibiting a large absorption coefficient >$10^{4}/$cm. However, the lattice mismatch with GaN limits the total InGaN thickness to a few tens of nanometers, resulting in a low absorption efficiency and photodiode responsivity.

To increase the light harvesting, one can employ light trapping structures, such as optical cavities, a roughened surface plus a back-reflector, plasmonic structures, and photonic crystals. In this work, we investigated a simple strategy using a metal-coated patterned sapphire substrate (pss) acting as a back-reflector and light scatterer to deflect the incident light in order to increase the optical path. The process of fabricating a pss is well established and widely used in the LED industry [31]. As a result, the proposed pss as a wafer-scale light trapping structure can be fairly economical to manufacture. In the current design, we consider the pattern to be fabricated on the bottom side of the sapphire substrate, as a laser lift-off (LLO) process to separate the GaN layer from the pss substrate is still in development [32]. In the future, it may be possible for the photodiode epitaxial stack to be grown directly on a pss, which can then be coated from the underside with a metal back-reflector after substrate removal. In addition to the back-reflector, we also included optional top TiO${}_{2}$ grating, which we found to further enhance light absorption in large-diameter nanopillar structures.

**Figure 1:**
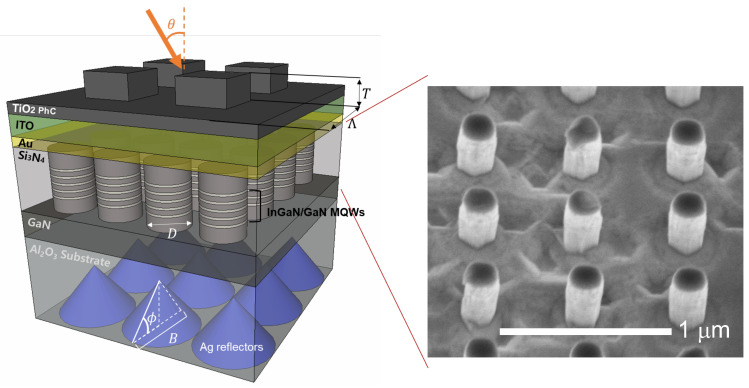
Schematic of the proposed wavelength-selective photodetector. The structure consists of three parts: the light-absorbing InGaN/GaN MQW active region, the photodetector p-type (ITO, Ni/Au) and n-type (not shown) contacts, and the light-trapping structure consisting of the silver (Ag)-coated cone-shaped back-reflector and the top TiO${}_{2}$ photonic crystal (PhC) layer. An unpolarized light incidents at an angle θ. The InGaN active region and the GaN pn junction are formed by lithographically patterning a thin-film structure of the same epitaxial stack, as shown by the scanning electron micrograph on the right, with an array of InGaN DIW nanopillars with a diameter of 200 nm before filling the space between the nanopillars with the insulating Si${}_{3}$N${}_{4}$.

Figure 1 shows the schematic of the proposed device structure. For the pss, we considered an array of cone-shaped, silver (Ag)-coated structures with adjustable sidewall angle ϕ, which can be tweaked by the resist reflow condition and the subsequent transferring of the resist pattern into the sapphire via dry etching [31]. We fixed the base diameter *B* at 1.2 μm and assumed the backside of the sapphire substrate was polished before the cone structures were formed. The photodiode consists of an array of nanopillars. The epitaxial stack of the nanopillar is a typical LED structure with an InGaN/GaN MQW active region sandwiched between a GaN pn junction. The nanopillar’s diameter *D* determines the active region’s absorption cut-off wavelength. In this work, we considered five pairs of 2.5 nm InGaN/12 nm GaN MQWs with an emission wavelength of 590 nm at room temperature. The absorption cut-off wavelength decreased with the nanopillar diameter and became 485 nm with a nanopillar diameter of 50 nm. To complete the photodiode, the nanopillars were first coated with 50 nm of SiN${}_{x}$ as the insulator and the void were filled and planarized by SiO${}_{2}$. The p-type contact consists of 5 nm each of Ni and Au plus a 200 nm-thick indium–tin–oxide (ITO) as the current spreading layer. The device fabrication and photodiode characterizations were previously reported in Ref. [28].

To evaluate the light-harvesting efficiency (LHE), which we defined as the fraction of light intensity absorbed by the InGaN active region in the wavelength range 20 nm above the cut-off wavelength, we used finite-difference time-domain (FDTD) simulations with a periodic boundary condition. We considered an unpolarized incident light at an angle θ. We included the entire epitaxial stack, including the metal layers, and assumed a transparent Ni layer. After thermal annealing of the p-type contact in air at a high temperature, the Ni layer transformed into a transparent NiO. Although the absorption in InGaN depends on the wavelength, we assumed a constant absorption coefficient of $10^{5}$ cm${}^{-1}$ for simplicity. This assumption is justified, because we are mainly interested in the relative improvement in absorption after introducing the light-trapping structure. We considered five different nanopillar diameters: 50 nm, 100 nm, 200 nm, 1000 nm, and thin film, corresponding to the absorption cut-off wavelengths of 485 nm, 518 nm, 536 nm, 570 nm, and 590 nm, respectively.

## 3. Results and Discussions

**Figure 2:**
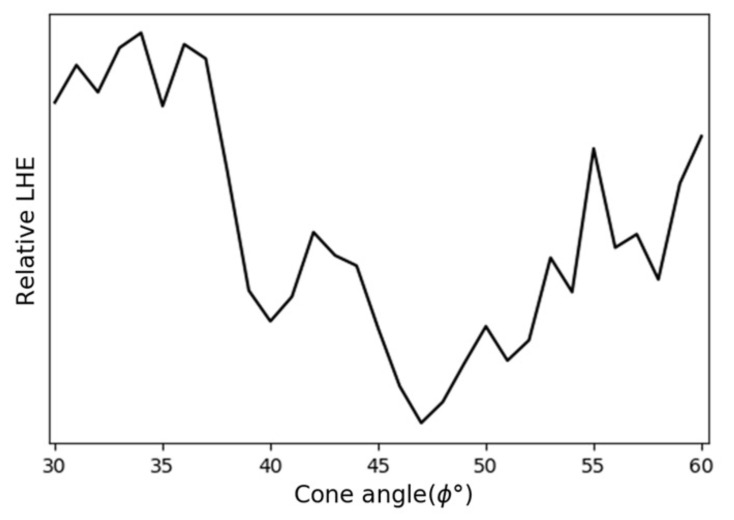
The relative LHE as a function of the cone-shaped Ag back-reflector’s sidewall angle ϕ calculated using 2D FDTD simulations. The nanopillar diameter is 50 nm. The edge-to-edge spacing between two adjacent pillars is 50 nm. The TiO${}_{2}$ grating is not included.

We first determined the optimal cone angle ϕ for the back-reflector. We focused on the smallest diameter nanopillar array, as it has the lowest LHE. The edge-to-edge spacing between two adjacent nanopillars was fixed at 50 nm for all nanopillar arrays. This spacing was chosen because it can be readily patterned using modern lithographic tools. Figure 2 compares the relative LHE as a function of the cone angle. For simplicity, we performed only 2D-FDTD calculations, as the goal was to determine the optimal cone angle rather than calculating the exact LHE. The cone’s base dimension was fixed at 1.2 μm. It can be seen that the LHE peaks at a cone angle range between 32° and 37°. We fix ϕ= 33° in the following discussions.

Next, we calculated the LHE for each photodetector and compared the result to the baseline, i.e., without the back-reflector, as well as the baseline plus a flat back-reflector. As a reference, we also calculated the LHE for a device structure that was previously demonstrated experimentally, which was nothing but the baseline with a much lower density of nanopillars.

**Figure 3:**
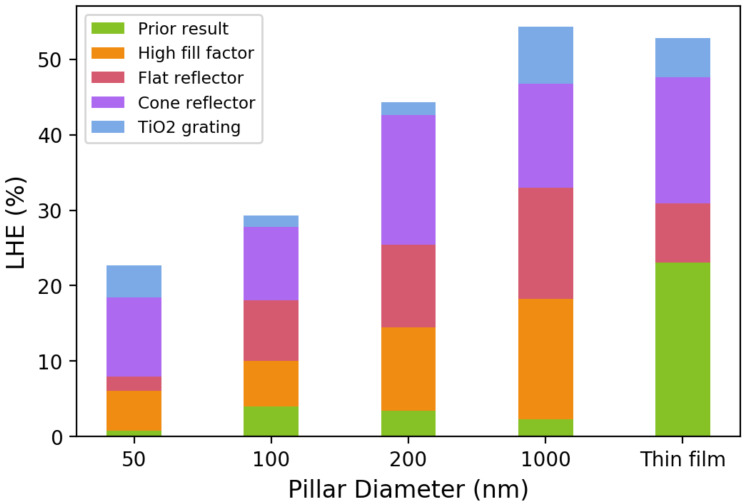
LHEs of various photodetectors with different nanopillar diameters for an unpolarized, normally incident light. The absorption cut-off wavelengths are 485 nm, 518 nm, 536 nm, 570 nm, and 590 nm in the order of the nanopillar diameter. The LHE is defined as the fraction of light intensity absorbed by the InGaN active region in the wavelength range 20 nm above the cut-off wavelength. For each photodetector, the LHEs for different light-trapping designs are also shown: “prior result” corresponds to the experimental device that was previously reported without any light-trapping structure and with a low nanopillar fill factor; “high fill factor” corresponds to a nanopillar array with an edge-to-edge spacing between two adjacent nanopillars; “flat reflector” corresponds to the coating of Ag on the polished bottom side of the sapphire substrate; “cone reflector” corresponds to the addition of a cone-shaped, Ag-coated back-reflector on the bottom side of the sapphire substrate; “TiO${}_{2}$ grating” corresponds to the complete light-trapping structure shown in Figure 1.

Figure 3 compares the LHE’s for different light-trapping designs for a normally incident light. We can see that the simple cone reflector increases the LHE for all detectors by roughly two to three times compared to the baseline. As the LHE increases, the photodetector’s responsivity and the spectral reconstruction’s accuracy also increase, as is discussed below. The cone reflector on the backside of the sapphire substrate can be easily and economically fabricated. However, the performance gain over a flat reflector is considerable. As light passes through the nanopillar array, light scattering occurs. The cone-shaped reflector further increases the light angle passing through the InGaN/GaN MQW region the second time, resulting in a long absorption path. Further improvements in LHE can be achieved by creating a third pass through the MQWs. We chose not to use optical cavities or other structures that can introduce wavelength dependence or/and incident angle sensitivity. Instead, we proposed to add a 200 nm TiO${}_{2}$ film on top of the ITO layer. The TiO${}_{2}$ film has an 83% transmittance at a 500 nm wavelength. To suppress the incident angle dependence, we added a two-dimensional photonic crystal (PhC) structure on top with a period of 200 nm and a duty cycle of 50% in both directions. The height of the PhC layer is 100 nm. It can be observed in Figure 3 that the top TiO${}_{2}$ layer further improves the LHE in all detectors. Compared to the previous design in Ref. [17], the proposed pss and the TiO${}_{2}$ grating in this work significantly enhance the LHE. As is discussed later, the improvement of nearly 20× in LHE for the 1000 nm diameter photodetector considerably improves the spectral reconstruction performance. It is worth noting that the TiO${}_{2}$ structure introduced here is uniform across all devices and was intentionally not optimized for the optical cavity effect. A high transmittance through TiO${}_{2}$ also ensures that the majority of the incident light can enter the structure.

**Figure 4:**
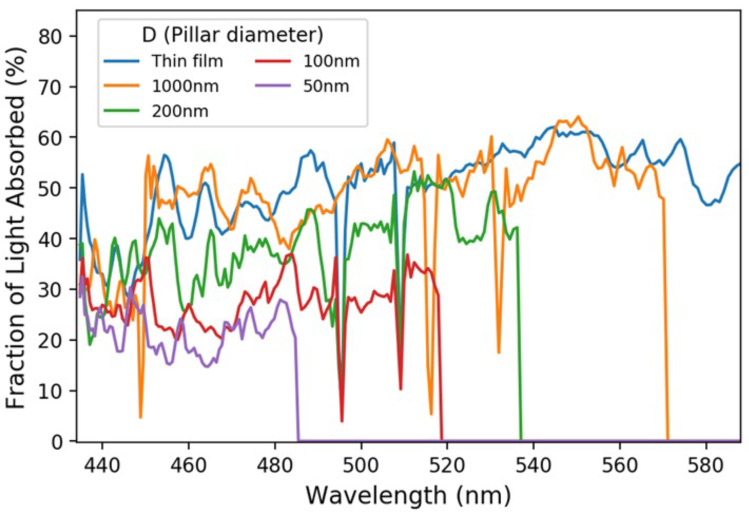
Broadband characteristics of the absorption responses of various photodetectors with different nanopillar diameters. The fraction of light absorbed in percentage as a function of the wavelength for each photodetector is shown. The structure of the photodetector is given in Figure 1. The absorption cut-off wavelengths are 485 nm, 518 nm, 536 nm, 570 nm, and 590 nm in the order of the nanopillar diameter starting from 50 nm. The incident light is unpolarized and has a 0° incident angle, i.e., normal to the detector surface.

The absorption responses of different photodetectors in an absorption-based spectrometer need to be uncorrelated. In our design, the correlation is removed by varying the absorption cut-off wavelength. Figure 4 shows the broadband characteristics of the absorption of various photodetectors with different nanopillar diameters. It is observed that the absorption responses are relatively flat with respect to the wavelength for all detectors. Hence, the degree of correlation being removed is mainly determined by the absorption of each photodetector in a narrow spectral band just above the cut-off wavelength, which is why we chose to benchmark our design by defining the LHE as the fraction of light absorbed in the 20 nm wavelength window above the cut-off wavelength. Figure 4 also suggests that the spectral reconstruction performance depends strongly on individual photodetectors’ LHEs. Increasing the LHEs can improve the spectral reconstruction, as is shown below.

**Figure 5:**
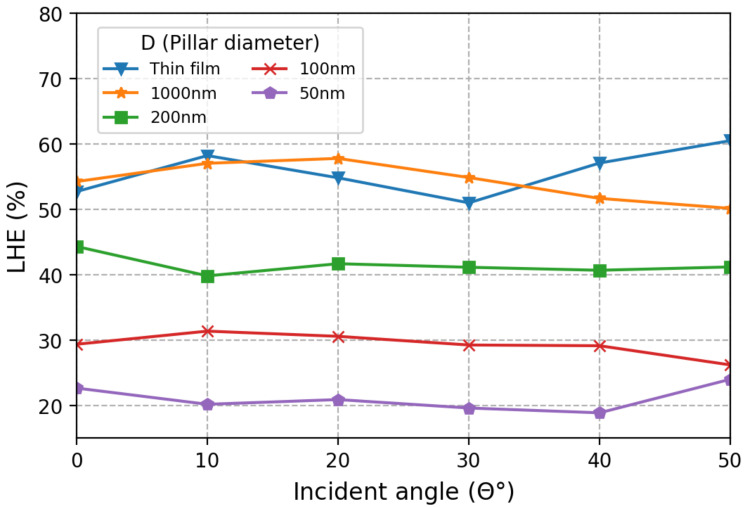
LHE as a function of the light’s incident angle θ. Photodetectors with small nanopillar diameters exhibit LHEs that are relatively insensitive to θ. Large-diameter photodetectors exhibit more angle dependence, which can be suppressed by the addition of a high-efficiency optical diffuser placed at the surface of the device. The absorption cut-off wavelengths are 485 nm, 518 nm, 536 nm, 570 nm, and 590 nm in the order of the nanopillar diameter.

Figure 5 shows the LHEs at different incident angles. We mainly focus on an incident angle from 0° to 50°, which corresponds a numerical aperture of 0.77. This should include most optics if they are to be used with the proposed photodetector array. One advantage of the absorption-based spectrometer compared to a DOE-based spectrometer is the incident angle insensitivity, which enables compact construction of the entire system, including the use of a high numerical aperture focusing lens. The introduction of light-trapping structures will inevitably introduce an unwanted angle dependence. As shown in Figure 5, the incident angle dependence is relatively weak for small-nanopillar detectors but not negligible for 1000 nm and thin-film devices. Even so, the angle dependence is not very strong and is expected to be easily mitigated using a high-efficiency optical diffuser.

Finally, we examined how the improved LHE impacts the spectrometer’s performance. We considered 14 photodetectors with absorption cut-off wavelengths varying from 485 nm to 590 nm. Starting from the experimentally measured responsivity curves for these detectors, we estimated the new responsivity matrix *R* based on the calculated LHEs. To compare the spectral reconstruction’s performance, we inputted a monochromatic light δ(λ), varied its wavelength λ from 460 nm to 590 nm, determined the theoretical photocurrents $I_{p}=R\times \delta \left(\lambda \right)$, and calculated the peak positions of the reconstructed spectra *P* using the non-negative least square (NNLS) algorithm with an $L_{1}$ norm $\left|\right|\cdot {\left|\right|}_{1}$ on the following inequality [13]:

(2) $$\min_{P\geq 0}\left|\right|I_{p}-RP{\left|\right|}_{1}^{2}.$$

**Figure 6:**
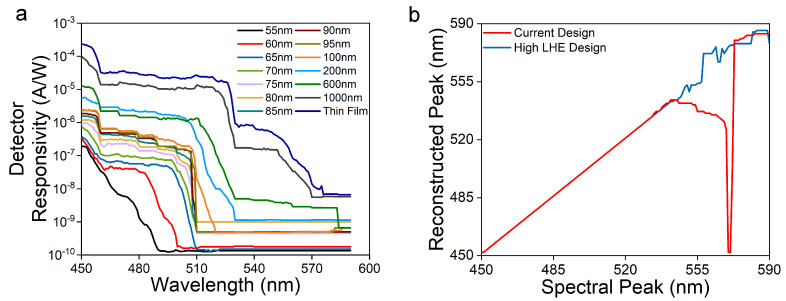
(**a**) Experimentally measured responsitivities of 14 photodetectors of different nanopillar diameters (data adapted from Ref. [17]). The LHEs of these devices are shown as “prior result” in Figure 3. (**b**) The spectral reconstruction performance determined using the responsivity matrix $R_{ij}$, where *i* and *j* correspond to the i−th photodetector and wavelength $\lambda_{j}$, respectively; the theoretical photocurrent $I_{i}$ of the i−th photodetector determined by $I_{i}=R\times \delta \left(\lambda \right)$, where δ is the Dirac delta function; and Equation (2). The “current design” corresponds to the responsivity matrix shown in (**a**). The “high LHE design” corresponds to the design shown in Figure 1.

The result is shown in Figure 6. Without the LHE enhancement, the spectral reconstruction has a large error between 540 nm and 570 nm. The cone reflector increases its LHE by more than 10 times, resulting in much more accurate spectral reconstruction. Further improvements are expected by increasing the number of photodetectors in the long wavelength range and the level of responsivity with improved electrical characteristics.

## 4. Conclusions

In summary, we have demonstrated an absorption-based on-chip optical spectrometer design based on a standard LED epitaxial stack. Using local strain engineering, the detector’s absorption cut-off wavelength can be tuned geometrically by various lithographically defined arrays of nanopillars with different diameters. In spite of InGaN’s high absorption coefficient, the lattice mismatch between InGaN and GaN limits the LHE of an InGaN/GaN MQWs to a few percentage points. To this end, we introduced a simple cone-shaped, Ag-coated back-reflector at the bottom side of the sapphire substrate to enhance the absorption. The interplay between the light scattering from the nanopillar array and the deflection from the cone shape significantly increase the absorption length through the active region. The LHE can be further enhanced with the addition of a thin TiO${}_{2}$ film dotted with a two-dimensional TiO${}_{2}$ periodic structure. Using a photodetector design previously demonstrated by experiments, LHEs of 20–60% can be obtained in the wavelength range of 450 nm–590 nm, which represents a 10-fold improvement on the prior results.

Not only can the Ag-coated cone-shape back-reflector be readily fabricated using mature patterned sapphire substrate processes, but the enhanced LHEs can lead to improved accuracy of spectral reconstruction in the situation when the number of photodetectors is smaller than the degree of freedom or the number of independent wavelengths. Moreover, the LHE enhancement to the cone-shaped back-reflector and the top TiO${}_{2}$ layer exhibit weak dependence on the incident angle of light, reducing the requirement to include collimation optics as is often needed for most spectrometers. Therefore, our spectrometer design can enable an ultrathin form factor for an array of spectrometers on the chip, which can be attractive for many mobile applications.
